# Congruent sonification during practice impairs cue-reduced recall in a virtual reality rhythm-game task

**DOI:** 10.3389/fpsyg.2026.1813641

**Published:** 2026-05-01

**Authors:** Ryota Sasaki, Arinobu Niijima, Chanho Park, Takefumi Ogawa

**Affiliations:** 1 The University of Tokyo, Tokyo, Japan; 2 NTT, Inc., Kanagawa, Japan

**Keywords:** motor learning, recall, rhythm-game, sonification, virtual reality

## Abstract

**Introduction:**

Rhythm-game learning naturally couples auditory and visual information, but always-available sensory cues may act as guidance and limit performance when those cues are withdrawn. We tested whether congruent sonification during practice strengthens a trajectory representation or instead promotes cue dependency in a virtual reality (VR) rhythm-game task.

**Methods:**

Forty right-handed participants learned a fixed 15-bar left/center/right trajectory sequence in VR. Across 24 sets, participants practiced with visual feedback only in the Control group or with congruent audiovisual feedback in the Sonification group. Each set comprised three feedback-rich practice trials followed by a silent memory-test trial in which the note stream and auditory feedback were removed. Performance was quantified by the accuracy (hit rate) and the practice-to-test decrement (Δhit rate).

**Results:**

Practice hit rate improved similarly in both groups and plateaued around 80%. In contrast, cue-reduced silent-test performance diverged late in training (sets 22–24): the Control group outperformed the Sonification group (hit rate: 81.9%±9.4% vs. 68.4%±18.4%, *p*_adj_ = 0.023, Cliff's δ = 0.495). The practice-to-test decrement also indicated greater cue dependency with sonification (Δhit rate: 1.6%±7.4% vs. −10.7%±18.5%, *p*_adj_ = 0.0044, Cliff's δ = 0.590).

**Discussion:**

Despite comparable acquisition, sound-guided practice reduced performance when auditory and visual cues were withdrawn, consistent with the guidance hypothesis and specificity-of-practice accounts. These results suggest that VR motor training should match the intended execution context or employ faded sonification schedules to promote robust retention.

## 1 Introduction

Rhythm games provide a compelling setting for studying and training sensorimotor skills that require precise temporal coordination. In rhythm-game-like tasks, performers synchronize movements to an externally structured rhythm while selecting among rapidly changing spatial targets, a form of sensorimotor synchronization that has been widely studied in the tapping and synchronization literature (Repp, 2005; Repp and Su, 2013). Virtual reality (VR) further amplifies the coupling between perception and action by embedding targets and actions in an immersive visuomotor space, and VR rhythm games have become a common testbed for studying how auditory–motor alignment influences performance and experience (Albert et al., 2022). Beyond entertainment, VR games and game-like tasks are increasingly explored as training and rehabilitation tools, including for upper-limb movement practice and quantification (Barclay et al., 2023; Demers et al., 2021).

At the same time, rhythm-game performance is typically scaffolded by rich, continuously available sensory cues (e.g., visual target streams, music, and additional action-linked sounds). This raises a fundamental motor-learning question: under such cue-rich practice, to what extent is the sequence internalized into an intrinsic sensorimotor representation, and how robustly can it be expressed when those external cues are reduced or withdrawn?

A central construct in motor-learning theory is the role of feedback beyond intrinsic sensory signals. Augmented feedback (information about task state or performance provided externally) can improve online control during practice by reducing uncertainty and supporting error correction (Salmoni et al., 1984; Schmidt, 1991). Auditory feedback is a particularly flexible form of augmentation. Here we focus on action-linked auditory feedback (sonification of the performer's state), which is conceptually distinct from background music or music–gameplay synchronization cues. Through movement sonification, action variables can be mapped to sound in real time, enabling learners to hear aspects of their movements such as position, velocity, or error. Recent reviews highlight the growing diversity of real-time movement sonification systems, especially in rehabilitation-oriented applications (Nown et al., 2022). In VR contexts where haptic information can be limited and visual displays can become overloaded, auditory channels are often proposed as a complementary route to increase informativeness and engagement (Demers et al., 2021). For example, in VR eyes-free interaction, sonification has been shown to improve the accuracy and perceived ease of selecting out-of-view targets by mapping spatial offsets to multiple auditory parameters (Takahara et al., 2025).

However, improved performance during practice does not necessarily imply improved learning. Classic accounts of augmented feedback emphasize a potential cost of concurrent, always-available guidance. According to the guidance hypothesis, learners may come to rely on augmented feedback for moment-to-moment control, thereby failing to develop a sufficiently robust internal representation to support performance when feedback is withdrawn (Salmoni et al., 1984; Schmidt, 1991). Consistent with this view, concurrent feedback can enhance acquisition-like performance yet impair retention relative to conditions that encourage internalization of task-relevant information (Sigrist et al., 2013). At the same time, contemporary syntheses suggest that the impact of feedback frequency on learning and performance can be heterogeneous and sometimes small, underscoring the need for task-specific tests that directly probe cue dependency (McKay et al., 2022).

For rhythm-game-like learning, auditory information can constitute task-relevant structure (timing, accent, and event identity) rather than a purely extrinsic cue (Repp and Su, 2013). On the other hand, when sound provides a direct, low-effort indicator of the required action (e.g., a discrete action-to-pitch mapping that transparently reveals the correct spatial state), it may function as continuous guidance and foster cue dependency. This possibility is compatible with reports that the behavioral consequences of augmented feedback depend on modality and how the signal is used for control (Ronsse et al., 2011).

Despite growing interest in sonification for motor training, relatively few studies directly test whether congruent auditory feedback during practice improves cue-reduced execution in tasks that require reproducing a learned movement sequence from memory. Existing work often focuses on online trajectory adjustments or short-term performance benefits with sonification (Barkasi et al., 2024), or on using real-time auditory cues to shape movement quality in clinical populations (Douglass-Kirk et al., 2023). Whether congruent sonification strengthens a memorized movement sequence or instead promotes cue dependency remains unresolved in rhythm-game-like VR tasks. This issue is practically important because VR training often provides rich multimodal guidance, whereas real-world execution contexts may be quieter, noisier, or otherwise cue-limited, making robust cue-reduced retention a critical design goal.

The present study addresses this gap by examining how sound-guided practice affects cue-reduced recall in a VR rhythm-game trajectory-learning task. Participants learned a fixed sequence of discrete lateral target positions synchronized to a rapid temporal structure. Practice occurred with either visual feedback only or congruent audiovisual feedback via a parameter-mapping sonification that linked discrete hand positions to discrete pitches. Learning was assessed using cue-reduced silent memory tests in which both the visual target stream and the auditory feedback were removed, requiring participants to reproduce the learned sequence from memory.

We hypothesized two competing outcomes. If congruent sonification primarily enriches task-relevant information and strengthens the sensorimotor representation, it should enhance cue-reduced recall. Alternatively, if sonification acts as continuous guidance, it should produce a larger practice-to-test decrement and lower cue-reduced performance. To adjudicate between these possibilities, we quantified accuracy during feedback-rich practice and cue-reduced memory tests across training and computed the practice-to-test decrement as an index of cue dependency.

## 2 Materials and methods

We investigated motor learning in a VR rhythm-game-like task in which hand movements were tightly coupled to auditory feedback. Participants were assigned to either a Control group (visual feedback only during feedback-rich trials) or a Sonification group (congruent auditory feedback during feedback-rich trials), and group differences were evaluated in cue-reduced silent memory-test trials. The study was approved by the Ethics Review Committee of the University of Tokyo (approval no. E2025ALS067).

### 2.1 Participants

Forty healthy, right-handed adults (29 male, 11 female; age range: 18–40 years) participated in the experiment. Participants were randomly assigned to either the Control group (15 male, 5 female; *M*_age_ = 27.6 years, *SD* = 7.0, range: 19–40) or the Sonification group (14 male, 6 female; *M*_age_ = 25.9 years, *SD* = 5.5, range: 18–40). All participants provided written informed consent prior to participation.

The target sample size was set at 40 participants (20 per group) with reference to prior sonification and motor-learning studies using between-subjects designs and overall sample sizes in a similar range (Dyer et al., 2017).

We recorded self-reported background experience relevant to the task. In the Control group, 16/20 participants reported prior sports experience, 9/20 reported musical instrument experience, and 12/20 reported rhythm-game experience; in the Sonification group, the corresponding numbers were 14/20, 15/20, and 11/20, respectively. Participants were randomly assigned to groups by simple random allocation, without stratification for sex or prior task-relevant background variables; consequently, these characteristics were not perfectly balanced across groups.

### 2.2 Apparatus

The experiment was conducted using a head-mounted display (Meta Quest 3, Meta Platforms Technologies, LLC, Menlo Park, CA, United States) with Touch Plus controllers connected to a Windows 11 PC (Intel Core i7-13620H, NVIDIA GeForce RTX 4050). A custom VR application was developed in Unity (Unity 6000.1.5f1), and the application was configured to operate at a display refresh rate of 72 Hz. The experimenter monitored the participant's first-person view on the PC display throughout the task. Auditory feedback was delivered via the HMD's built-in speakers.

### 2.3 Task

**Figure 1:**
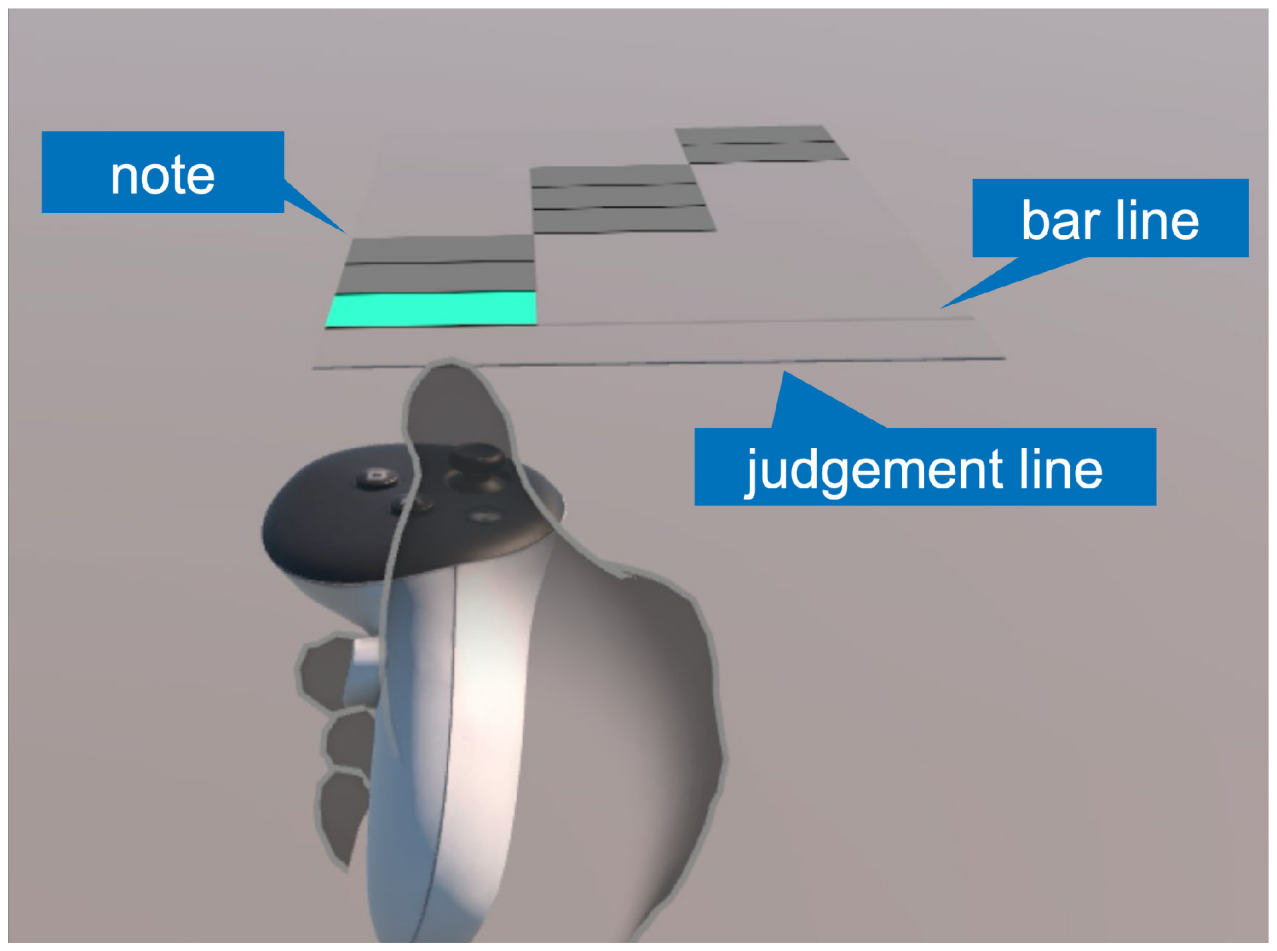
Screenshot of the VR rhythm-game trajectory-learning task. Rectangular targets (notes) approach the judgment line at a constant speed. Participants move the right-hand controller horizontally to the left/center/right position corresponding to each note when it crosses the judgment line. Bar-line markers provide temporal landmarks.

We developed a VR rhythm-game trajectory-learning task in which participants learned a fixed sequence of lateral hand positions synchronized to an approaching target stream. A single trajectory pattern was used throughout the experiment (i.e., an identical sequence was presented in all sets and trials). The stimulus consisted of rectangular targets (notes) that moved toward the participant (from far to near) at a constant speed of 200 cm/s and crossed a judgment line (Figure 1). Each note appeared at one of three discrete horizontal positions (left, center, or right) and was rendered as a rectangle (6 cm wide × 4 cm high). Participants stood while holding the VR controller and were instructed to move the right-hand controller horizontally to the corresponding position when a note reached the judgment line. A response was registered as a hit if, at any time during the interval in which a note overlapped the judgment line, the controller was within the corresponding target region. Thus, the spatial tolerance was defined by the rectangular note area, and the temporal tolerance corresponded to the duration of note–judgment-line overlap. Hits were indicated by an on-screen color change of the note.

The target stream comprised 360 notes (15 bars × 24 notes per bar), where a bar was defined as a fixed-length segment of 24 consecutive notes. Each bar lasted 480 ms, yielding a total sequence duration of 7.2 s. Importantly, all 24 notes within a bar shared the same horizontal position; thus, participants did not need to respond to 360 independent note events, but rather to 15 bar-level position events, with position changes occurring only at bar boundaries (i.e., one position every 480 ms). To provide temporal structure, bar-line markers were displayed at each bar boundary, serving as temporal landmarks analogous to measure lines in music notation. At any time, only the nine notes closest to the judgment line were rendered to limit visual load. Accordingly, the spatial component of the sequence can be summarized at the bar level as a 15-element position sequence (left/center/right).

The trajectory used in the present experiment was informed by prior preliminary work. Candidate movement patterns were first screened in a small preliminary test, and learnability was subsequently confirmed in a separate exploratory study with 24 participants who did not take part in the present experiment. In that exploratory study, participants completed two successive sets of a closely related memory-reproduction task, and reproduction-task hit rate was significantly higher in the second set than in the first, indicating that the sequence was learnable within a short training period. Accordingly, the present experiment used three observation–practice cycles per set and 24 sets in total to allow learning to be tracked over repeated practice while keeping the session duration manageable. A single trajectory pattern was then used throughout the experiment (i.e., the identical sequence was presented in all sets and trials) to reduce variability in sequence difficulty across repeated training and testing.

The task included two trial types: *practice* trials and *silent memory-test* trials. In practice trials, the note stream was visible and participants received online visual feedback for hits. In the Sonification group, a continuous pure-tone sonification was additionally presented during practice, with pitch mapped to the participant's right-hand position (left/center/right). In the Control group, practice was performed with sound muted. In silent memory-test trials, the note stream was removed (only the bar lines remained as temporal landmarks), and participants reproduced the learned movement sequence from memory. No auditory feedback was provided during silent memory-test trials in either group.

The experiment employed a between-subjects design with two training conditions that differed in the availability of auditory feedback during feedback-rich trials. Visual stimuli were identical across groups. In the Sonification condition, a parameter-mapping sonification linked the three discrete horizontal positions (left, center, right) to three pure-tone pitches (261.626, 293.665, and 392.628 Hz, respectively). In the Sonification group, prior to the task, participants adjusted the overall auditory feedback level once using an in-VR slider to a comfortable and clearly discriminable volume; this setting was then kept constant throughout the experiment.

### 2.4 Procedure

**Figure 2:**
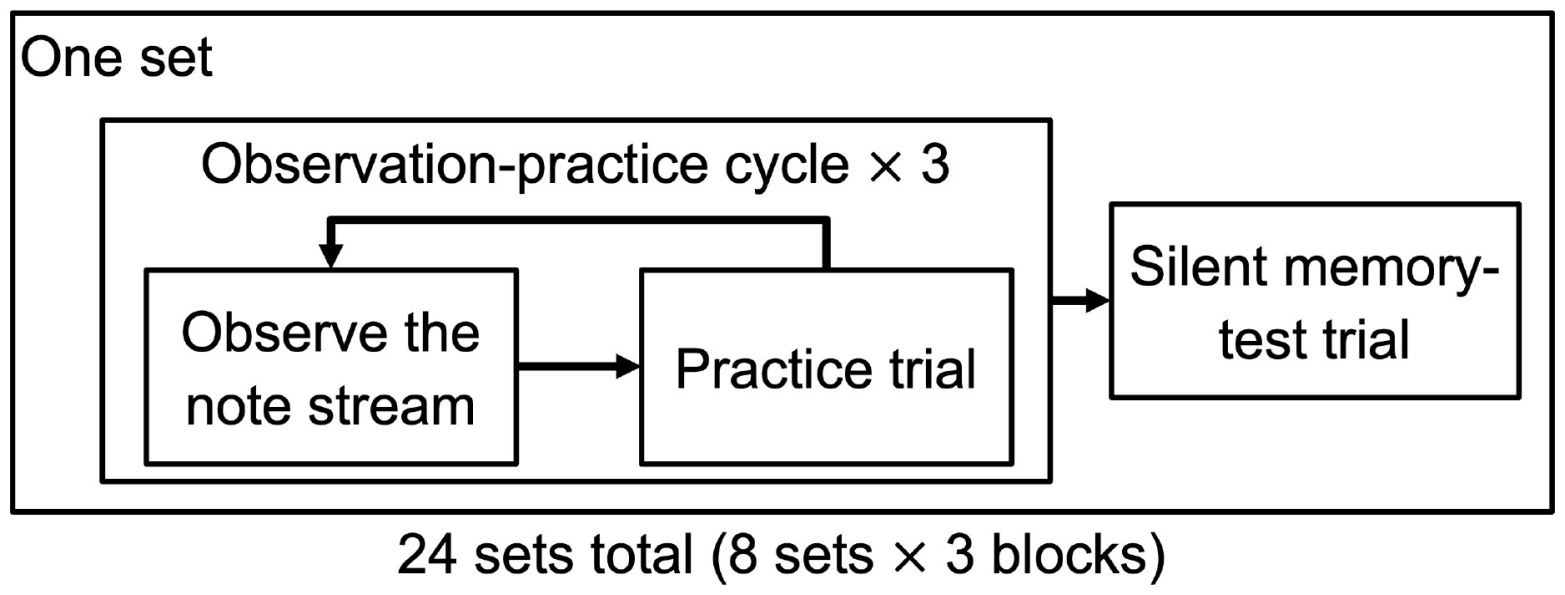
Schematic of the experimental procedure. One set consisted of three observation–practice cycles followed by one silent memory-test trial. The main session comprised 24 sets organized into three blocks of eight sets. Trajectory-drawing tests were administered after sets 8, 16, and 24. During the silent memory-test trial, the note stream and auditory feedback were removed, and only bar lines remained visible as temporal landmarks.

The main session comprised 24 sets organized into three blocks of eight sets, with short breaks between blocks (Figure 2). Participants stood while wearing the HMD and holding the controllers, and practiced the same fixed 15-bar sequence throughout the session using the right-hand controller. One set had the following structure: three observation–practice cycles, followed by one silent memory-test trial. Thus, each participant completed 72 practice trials and 24 silent memory-test trials in total.

In each observation–practice cycle, participants first observed the note stream from start to end. In the Sonification group, during the observation phase, participants heard a pre-recorded auditory preview of the correct sequence, temporally aligned with the note stream and generated using the same left/center/right pitch mapping as the real-time sonification used during practice. Participants then performed one practice trial, reproducing the sequence in synchrony with the incoming notes. During practice trials, the note stream was visible in both groups, whereas real-time auditory feedback was additionally provided only in the Sonification group. After the third observation–practice cycle, participants performed one silent memory-test trial, in which the note stream and auditory feedback were removed and only the bar lines remained visible as temporal landmarks. During the breaks after sets 8, 16, and 24, participants additionally performed a trajectory-drawing task to report the remembered left/center/right position for each of the 15 bars. After each trial, participants in both groups were shown the resulting hit rate in VR as terminal feedback before proceeding to the next trial. This was intended as minimal knowledge of results, particularly for the silent memory-test trials, in which participants could not readily evaluate performance because the note stream and auditory feedback were absent.

Note that, in the Sonification group, auditory feedback during practice was action-contingent rather than note-triggered: a pure tone corresponding to the participant's current left/center/right hand position was generated online as a function of the current controller position. Thus, the auditory signal was temporally linked to the participant's movement, not to scheduled note onsets. During observation, by contrast, participants heard an ideal reference tone sequence corresponding to perfect performance and aligned with the visual note stream. End-to-end system latency was not independently quantified in the present setup.

In silent memory-test trials, the underlying target stream had the same temporal structure and duration as in practice trials (360 notes over 7.2 s; 15 bars, 480 ms per bar), but the note stream and auditory feedback were removed. Participants reproduced the sequence in real time using only the bar lines as temporal landmarks. Hit scoring was identical to that in practice trials.

### 2.5 Analysis

All analyses were planned to compare acquisition during practice with cue-reduced performance during silent memory-test trials between the Control and Sonification groups. Our primary goal was to compare the two groups at distinct learning stages for acquisition performance, cue-reduced test performance, and practice-to-test decrement. To obtain interpretable learning-stage measures while reducing trial-to-trial and set-to-set variability, we summarized performance within predefined early, mid, and late windows, yielding one window-level value per participant for each outcome.

#### 2.5.1 Hit-rate measures

Task performance was quantified as hit rate for each trial. For each set, we computed (1) *practice hit rate* as the mean hit rate across the three practice trials and (2) *test hit rate* as the hit rate in the subsequent silent memory-test trial. To quantify the cost of removing external cues, we defined the *practice-to-test decrement* as


Δhit rate=(test hit rate)-(practice hit rate),

where more negative values indicate a larger drop in performance from practice to the cue-reduced test.

#### 2.5.2 Planned-window averaging

To obtain interpretable learning-stage measures and reduce set-to-set variability, we averaged set-wise performance within three pre-defined windows: early (sets 1–3), mid (sets 11–13), and late (sets 22–24). This yielded one window-level value per participant for each outcome (practice hit rate, test hit rate, and Δhit rate).

#### 2.5.3 Omnibus and planned-window comparisons

To provide an omnibus evaluation of group differences across learning stages, we first analyzed each outcome (practice hit rate, test hit rate, and Δhit rate) using a two-way mixed ANOVA with Group (Control vs. Sonification) as a between-subject factor and Window (early: sets 1–3; mid: sets 11–13; late: sets 22–24) as a within-subject factor. For each ANOVA, we tested the main effects of Group and Window and the Group × Window interaction. Effect sizes are reported as partial eta squared ($\eta_{p}^{2}$).

We then compared groups (Control vs. Sonification; independent samples) separately within each planned window. Normality was assessed using the Shapiro–Wilk test within each Group × Window cell. If neither group showed evidence of deviation from normality (*p*>0.05 in both groups), we used Welch's two-sample *t*-test; otherwise, we used the two-sided Mann–Whitney *U* test. Effect sizes were reported as Hedges' *g* for *t*-tests and Cliff's δ for Mann–Whitney tests. For each outcome, *p*-values across the three planned windows were adjusted using the Bonferroni correction.

#### 2.5.4 Trajectory-drawing memory test

Trajectory memory was assessed using the trajectory-drawing score (0–15) collected at three timepoints corresponding to the breaks after sets 8, 16, and 24. Group differences at each timepoint were evaluated using Welch's *t*-tests or Mann–Whitney *U* tests (based on Shapiro–Wilk normality checks), with effect sizes reported as Hedges' *g* or Cliff's δ, respectively. The *p*-values across the three timepoints were adjusted using the Bonferroni correction.

## 3 Results

All participants completed the experiment and were included in the hit-rate analyses.

### 3.1 Set-by-set learning curves

**Figure 3:**
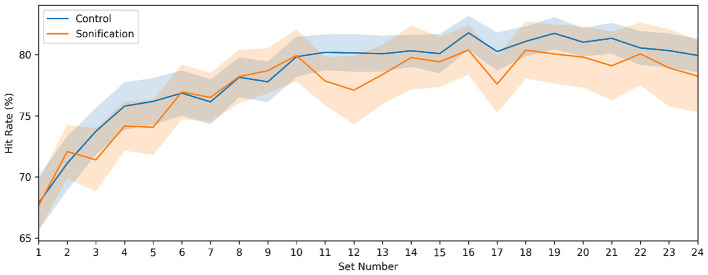
Set-by-set practice performance. Practice hit rate averaged across the three practice trials in each set for the Control and Sonification groups. Lines show group means across participants and shaded bands indicate standard error of the mean.

Figure 3 shows the set-wise practice hit rate (mean across the three practice trials in each set; group mean ± SEM, where SEM denotes the standard error of the mean). Practice performance improved over sets in both groups and approached an asymptote near the end of training, with broadly similar learning trajectories.

**Figure 4:**
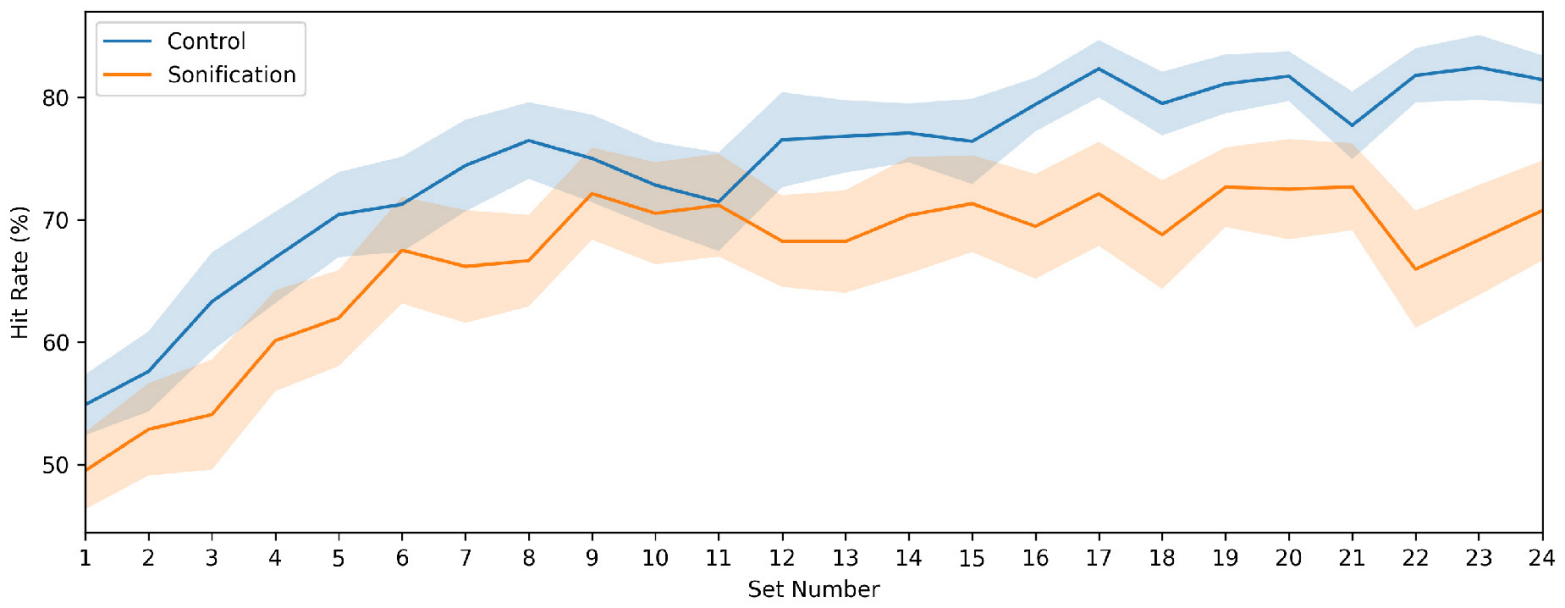
Set-by-set cue-reduced silent-test performance. Test hit rate for the silent memory-test trial at the end of each set, in which the note stream and auditory feedback were removed. Lines show group means and shaded bands indicate standard error of the mean.

Figure 4 shows the set-wise silent memory-test hit rate (group mean ± SEM). Silent-test performance increased over time in both groups, but the trajectories diverged in later sets: the Control group continued to improve, whereas the Sonification group showed a shallower increase and tended to plateau.

**Figure 5:**
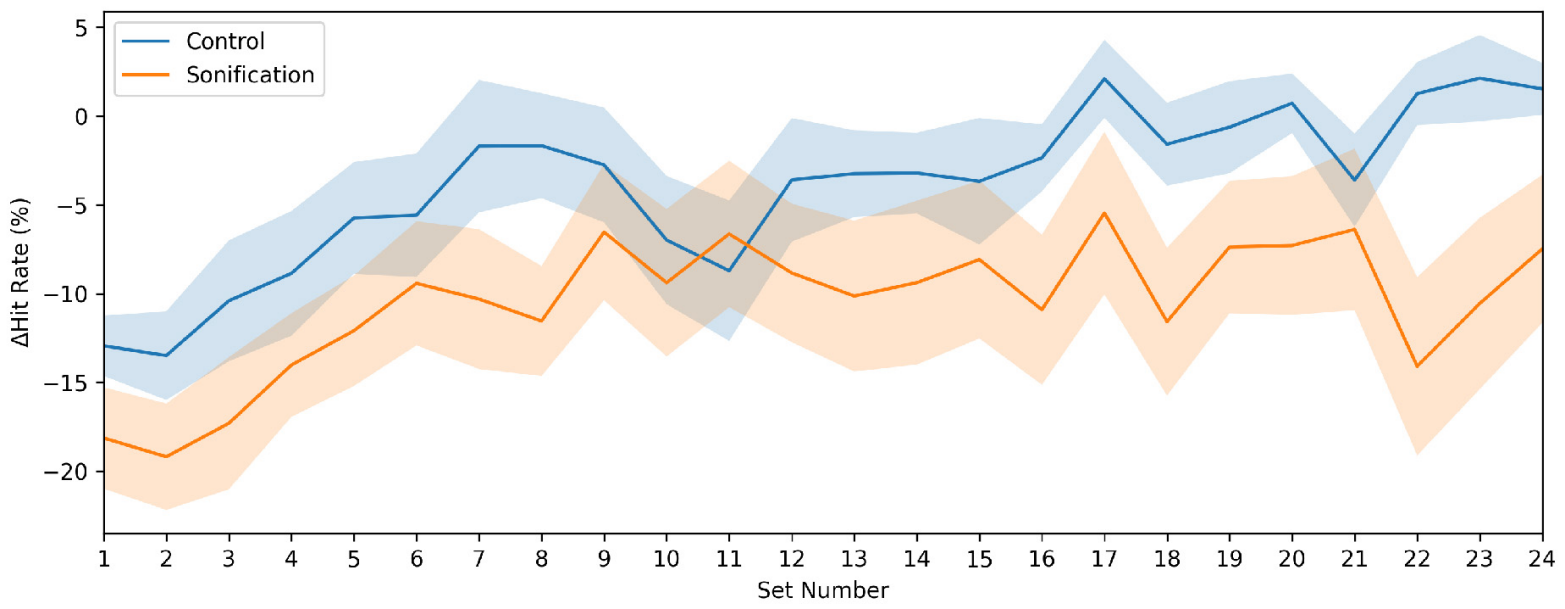
Set-by-set practice-to-test decrement (Δhit rate). Negative values indicate a drop in performance when external cues were removed. Lines show group means and shaded bands indicate standard error of the mean.

Figure 5 shows the set-wise practice-to-test decrement, Δhit rate (group mean ± SEM). Across sets, Δhit rate became progressively less negative in the Control group, approaching near-zero (and occasionally positive) values in later sets. In contrast, Δhit rate remained negative in the Sonification group throughout training, indicating a persistent performance drop when external cues were removed.

### 3.2 Planned-window comparisons

To summarize learning-stage performance and statistically compare groups, we first conducted two-way mixed ANOVAs for practice hit rate, test hit rate, and Δhit rate, with Group (Control vs. Sonification) as a between-subject factor and Window (early: sets 1–3; mid: sets 11–13; late: sets 22–24) as a within-subject factor. We then performed planned window-specific between-group comparisons to localize differences across learning stages. Unless otherwise noted, window-level values are reported as mean ± SD across participants. For the planned comparisons, Control and Sonification were compared using Welch's *t*-tests or Mann–Whitney U tests as appropriate (based on Shapiro–Wilk normality checks), with Bonferroni correction across the three windows for each outcome.

#### 3.2.1 Practice hit rate

A two-way mixed ANOVA on practice hit rate, with Group as a between-subject factor and Window as a within-subject factor, showed a significant main effect of Window (*F*(2, 76) = 39.66, *p* < 0.001, $\eta_{p}^{2}=0.511$), but no main effect of Group (*F*(1, 38) = 0.27, *p* = 0.606, $\eta_{p}^{2}=0.007$) and no Group × Window interaction (*F*(2, 76) = 0.34, *p* = 0.712, $\eta_{p}^{2}=0.009$).

**Figure 6:**
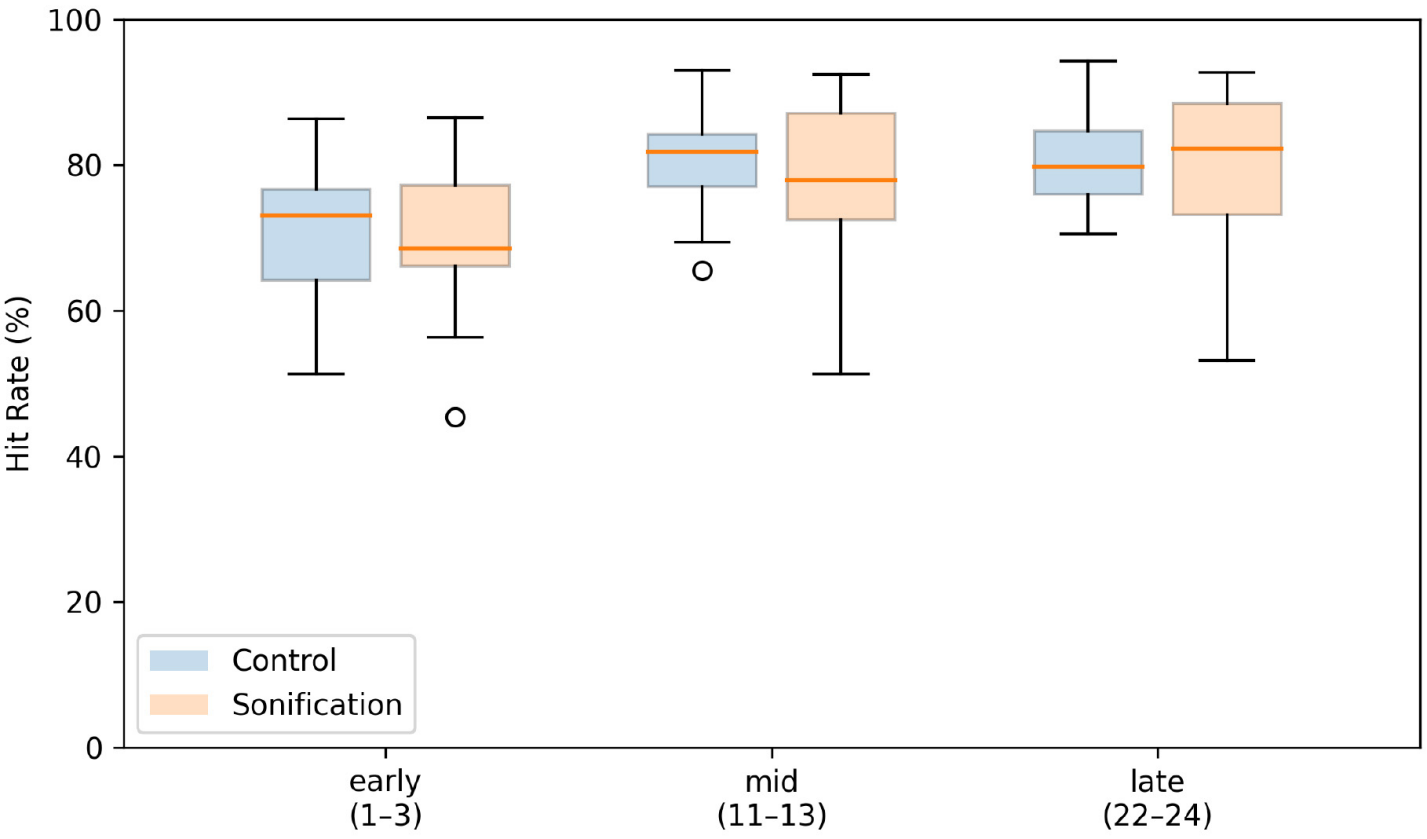
Planned-window averages of practice hit rate. For each participant, practice hit rate (mean across the three practice trials per set) was averaged within predefined windows: early (sets 1–3), mid (sets 11–13), and late (sets 22–24). Boxplots show the median (center line), interquartile range (box), whiskers (1.5 × IQR), and outliers (circles) for each group.

Planned-window averages of practice hit rate increased from early to mid and then plateaued in both groups (Figure 6). In the Control group, practice hit rate was 70.9%±8.8% (early), 80.1%±6.4% (mid), and 80.3%±6.0% (late). In the Sonification group, practice hit rate was 70.4%±9.7% (early), 77.8%±10.5% (mid), and 79.1%±12.4% (late).

#### 3.2.2 Test hit rate

A two-way mixed ANOVA on test hit rate showed significant main effects of Group (*F*(1, 38) = 4.44, *p* = 0.042, $\eta_{p}^{2}=0.105$) and Window (*F*(2, 76) = 60.52, *p* < 0.001, $\eta_{p}^{2}=0.614$), but no significant Group × Window interaction (*F*(2, 76) = 2.50, *p* = 0.089, $\eta_{p}^{2}=0.062$).

**Figure 7:**
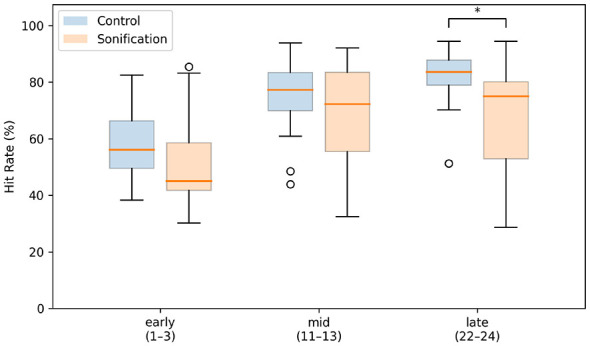
Planned-window averages of cue-reduced silent-test hit rate. For each participant, test hit rate from the silent memory-test trials was averaged within early (sets 1–3), mid (sets 11–13), and late (sets 22–24) windows. Boxplots show the median (center line), interquartile range (box), whiskers (1.5 × IQR), and outliers (circles) for each group. The asterisk indicates a significant difference.

Silent memory-test performance improved across windows in both groups, but differed in the late window (Figure 7). In the Control group, test hit rate increased from 58.6%±12.7% (early) to 75.0%±13.2% (mid) and 81.9%±9.4% (late). In the Sonification group, it increased from 52.2%±15.5% (early) to 69.2%±17.1% (mid), but remained lower in the late window (68.4%±18.4%).

Between-group comparisons indicated no significant differences in the early window (Mann–Whitney *U* = 266, *p*_adj_ = 0.229, Cliff's δ = 0.330) and mid window (Welch's *t*(35.6) = 1.19, *p*_adj_ = 0.731, Hedges' *g* = 0.367). In contrast, in the late window, the Control group significantly outperformed the Sonification group (Mann–Whitney *U* = 299, *p*_adj_ = 0.023, Cliff's δ = 0.495).

#### 3.2.3 Practice-to-test decrement

A two-way mixed ANOVA on Δhit rate showed significant main effects of Group (*F*(1, 38) = 5.00, *p* = 0.031, $\eta_{p}^{2}=0.116$) and Window (*F*(2, 76) = 12.50, *p* < 0.001, $\eta_{p}^{2}=0.248$), but no significant Group × Window interaction (*F*(2, 76) = 2.12, *p* = 0.128, $\eta_{p}^{2}=0.053$).

**Figure 8:**
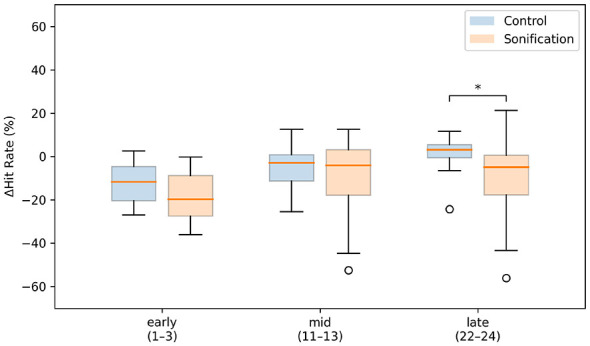
Planned-window averages of practice-to-test decrement (Δhit rate). For each participant, Δhit rate was averaged within early (sets 1–3), mid (sets 11–13), and late (sets 22–24) windows. Boxplots show the median (center line), interquartile range (box), whiskers (1.5 × IQR), and outliers (circles) for each group. The asterisk indicates a significant difference.

The practice-to-test decrement (Δhit rate) quantified the performance change when external cues were removed (Figure 8). In the early window, Δhit rate was negative in both groups (Control: −12.3%±9.0%; Sonification: −18.2%±11.8%), with no significant difference (Welch's *t*(35.5) = 1.79, *p*_adj_ = 0.247, Hedges' *g* = 0.554). In the mid window, Δhit rate remained negative in both groups (Control: −5.2%±11.1%; Sonification: −8.5%±17.0%), with no significant difference (Mann–Whitney *U* = 206, *p*_adj_ = 1.00, Cliff's δ = 0.030).

In the late window, however, the Control group showed a near-zero to slightly positive Δhit rate (1.6%±7.4%), whereas the Sonification group showed a clear negative Δhit rate (−10.7%±18.5%), indicating a larger practice-to-test decrement in the Sonification group (Mann–Whitney *U* = 318, *p*_adj_ = 0.0044, Cliff's δ = 0.590).

### 3.3 Trajectory-drawing memory test

Trajectory memory was assessed using the trajectory-drawing score (0–15) at three timepoints corresponding to the breaks after sets 8, 16, and 24. Scores increased over time in both groups and approached ceiling by the later timepoints (median = 15 after sets 16 and 24 in both groups).

Between-group comparisons at each timepoint showed no significant differences: after set 8 (Control: 9.70 ± 5.16, Sonification: 12.35 ± 4.02; Mann–Whitney *U* = 136.5, *p*_adj_ = 0.220, Cliff's δ = −0.318), after set 16 (Control: 13.50 ± 3.09, Sonification: 13.25 ± 2.90; Mann–Whitney *U* = 210.0, *p*_adj_ = 1.00, Cliff's δ = 0.050), and after set 24 (Control: 13.20 ± 3.87, Sonification: 14.15 ± 1.84; Mann–Whitney *U* = 186.0, *p*_adj_ = 1.00, Cliff's δ = −0.070).

## 4 Discussion

The present study asked whether congruent auditory feedback (sonification) during practice strengthens memory-based reproduction of a learned trajectory sequence, or instead promotes reliance on online task cues available during practice (i.e., the visible note stream and, in the Sonification group, auditory feedback) such that performance declines when these cues are reduced at test. Across 24 training sets, practice hit rate improved similarly in both groups and plateaued near 80%. In contrast, cue-reduced silent memory-test performance diverged late in training: the Control group achieved higher late-window test hit rates, and the Sonification group showed a larger practice-to-test decrement (Δhit rate). Together, these results indicate that, in the present design, the Sonification group showed poorer late cue-reduced timed execution, as reflected in lower late-window test hit rates and a larger practice-to-test decrement, despite similar trajectory-drawing performance. This pattern is consistent with the possibility that the added auditory information increased cue dependency.

Regarding the two competing hypotheses outlined in the Introduction, the present results are more consistent with the guidance account than with the idea that congruent sonification strengthened the sensorimotor representation in a way that benefited cue-reduced recall. If sonification had enhanced a sensorimotor representation robust to cue reduction, the Sonification group should have shown superior performance in the silent memory tests. Instead, the larger practice-to-test decrement and lower late-stage silent-test performance suggest that participants increasingly incorporated the sound cue into the practiced control strategy. Thus, sonification appears to have supported performance primarily when auditory information was available, rather than improving the cue-reduced sensorimotor representation required for silent execution. Importantly, this does not imply that no sequence representation was learned; rather, the benefit did not transfer to the representational and execution processes required for rapid cue-reduced performance.

### 4.1 Sound-guided practice reduced cue-reduced recall despite comparable acquisition

The dissociation between feedback-rich practice performance and cue-reduced test performance is consistent with classic accounts of augmented feedback. A long-standing view is that concurrent, always-available augmented feedback can function as *guidance*: it supports moment-to-moment control during practice but encourages reliance on the external signal, thereby degrading performance when the signal is removed (Salmoni et al., 1984; Schmidt, 1991). In the present task, sonification provided a low-effort state cue by mapping each discrete lateral position to a discrete pitch. Even if such feedback does not increase practice accuracy beyond a visually guided baseline, it can still alter the information learners prioritize for sequencing and state estimation, yielding a larger cost when that information is unavailable at test. More specifically, participants in the Sonification group may have increasingly weighted the auditory signal as an efficient cue for online sequence tracking, because the pitch mapping transparently labeled the current left/center/right state. This would reduce the need to rely on internally maintained state estimates during practice. Consequently, when the auditory channel was removed in the cue-reduced silent tests, retention of effective timed performance would be poorer, yielding a larger late practice-to-test decrement.

This interpretation aligns with work discussing auditory augmentation through the lens of the guidance hypothesis (Kohl and Shea, 1995). It is also compatible with evidence that real-time sonification can change online control policies, for example by inducing within-movement adjustments and shaping the kinematics used during acquisition (Barkasi et al., 2024). In our data, the persistent negative Δhit rate in the Sonification group suggests that practice with sound promoted a control strategy that was less robust when both the visual target stream and auditory feedback were absent.

At the same time, the present findings should not be interpreted as a general claim that sonification necessarily harms learning. Some studies report that appropriately structured sonification can support learning and can be retained when feedback is removed, suggesting that guidance effects depend on mapping design and task demands (Dyer et al., 2017). Likewise, in timing-focused paradigms, auditory feedback can support error-based learning and may transfer to mute testing when the auditory signal is informative about timing errors rather than merely substituting for state information (van Vugt and Tillmann, 2015). One implication is that our discrete state-to-pitch mapping may have been especially prone to cue binding because it transparently revealed the currently required spatial state with minimal inferential effort.

A complementary explanation is specificity of practice / encoding specificity: retention and transfer are best when the sensory context at test matches the context experienced during learning (Proteau et al., 1992; Tulving and Thomson, 1973). From this perspective, the Sonification group practiced (and observed) with an additional modality that was absent during the cue-reduced tests. With extended training, learners may increasingly weight the most salient or reliable channel for sequence tracking; consequently, removing that channel late in learning can reveal a larger context-mismatch cost. The late emergence of group differences is compatible with a gradual shift toward a sound-weighted strategy as practice progressed.

### 4.2 Trajectory-drawing memory and the dissociation between “knowing” and “doing”

Trajectory-drawing scores improved and approached ceiling in both groups, with no reliable group differences. This suggests that both groups acquired substantial explicit knowledge of the bar-level left/center/right sequence. The presence of a group difference in cue-reduced timed execution despite a null effect on explicit trajectory reports is consistent with the idea that sequence performance depends on multiple representational formats and execution processes (Verwey, 2001; Verwey et al., 2015; Willingham, 1999). In our task, the trajectory-drawing test emphasizes bar-level spatial order and is relatively insensitive to the continuous timing demands and rapid state-estimation requirements of producing 360 hits in synchrony. Thus, sonification may have left explicit sequence knowledge intact while still reducing the stability of rapid cue-reduced execution.

Importantly, this dissociation should be interpreted as a group-level tendency rather than as a uniform pattern across all individuals. In the late window, the Sonification group showed descriptively larger between-participant variability in cue-reduced performance than the Control group, both in test hit rate and in practice-to-test decrement. This suggests that the extent to which participants came to rely on auditory information may have differed across individuals, even though explicit bar-level sequence report was similar across groups.

Because the trajectory-drawing task required explicit report of the 15-bar left/center/right sequence during the breaks, it may have served as an intermittent rehearsal opportunity for both groups. However, this opportunity was identical across groups. Thus, although the task may have promoted explicit reflection on the sequence, it does not explain why the Sonification group still showed poorer late cue-reduced performance despite similar, near-ceiling trajectory-drawing scores. This pattern further supports a dissociation between explicit bar-level sequence knowledge and the rapid timed execution required in the silent memory-test trials.

### 4.3 Implications for sonification design in VR motor training

From an applied perspective, the findings caution against assuming that adding congruent sonification during practice will necessarily improve learning outcomes, particularly when the target execution environment is silent or otherwise cue-reduced. Two design implications follow.

First, practice should match the intended performance context; otherwise, specificity-of-practice costs may limit retention and transfer (Proteau et al., 1992; Tulving and Thomson, 1973). Second, when sonification is used, it may be beneficial to avoid continuous, always-on guidance throughout extended practice (Salmoni et al., 1984; Schmidt, 1991; Sigrist et al., 2013). In the present data, the between-group difference emerged late rather than early, suggesting that cue dependency may become more consequential as practice progresses. Accordingly, a plausible design strategy is to provide richer auditory support early in learning and then progressively reduce it later in training, for example, by fading its availability across sets, presenting it intermittently, or restricting it to terminal feedback after selected trials. Although the present study did not directly compare such schedules, these approaches may help limit sound dependency while preserving the informational benefits of sonification. This recommendation is consistent with feedback-scheduling work showing benefits of faded feedback protocols for retention and transfer in other motor domains (Aoyagi et al., 2019; Sato-Klemm et al., 2026).

More broadly, rhythm-game and exergame studies show that auditory–motor alignment can affect performance and experience during gameplay (Albert et al., 2022), but such work rarely tests whether audio-supported practice improves later execution when cues are withdrawn. Our results highlight the importance of evaluating sonification not only by acquisition performance but also by cue-reduced retention tests that reflect the intended execution setting.

### 4.4 Limitations

Several limitations warrant consideration. First, the current task used three discrete lateral positions and a single fixed trajectory throughout the experiment. Although this design reduced variability in sequence difficulty, it may also have promoted sequence-specific learning. Accordingly, the present findings may not generalize to other trajectories, mappings (e.g., error-based sonification), continuous trajectories, or richer action spaces. Second, retention was assessed within-session using repeated cue-reduced tests; future work should include delayed retention (e.g., 24 h, 1 week) and transfer tests to more cleanly separate transient performance effects from durable learning (Salmoni et al., 1984). Third, the trajectory-drawing test exhibited ceiling effects at later timepoints, limiting sensitivity to detect group differences in explicit memory. Fourth, because bar lines remained visible as temporal landmarks during the silent memory-test trials, retention was assessed under cue-reduced rather than fully cue-free conditions. These remaining temporal cues likely supported the timing of state transitions and reduced temporal memory demands. Accordingly, the present findings should be interpreted as reflecting cue-reduced retention of the learned spatial-state sequence under externally scaffolded timing, rather than fully self-generated reproduction of both timing and spatial-state information. Because the same temporal landmarks were available to both groups, this limitation does not directly account for the between-group difference. Future studies should manipulate or remove these landmarks to dissociate memory for when to switch from memory for where to move.

Another limitation of the present study is that the sample was male-biased (29 male, 11 female), because participants were recruited without sex-based stratification. The present study was not designed to evaluate sex-related effects, so the influence of sex on performance remains uncertain. In addition, participants were assigned without stratification for prior task-relevant background experience, and these backgrounds were therefore not perfectly balanced across groups, which may have contributed to inter-individual variability. A further methodological limitation is that end-to-end system latency and numerical tracking accuracy were not independently quantified in the present setup, so the extent to which device-level timing and tracking characteristics may have influenced performance remains uncertain. In addition, post-trial hit-rate feedback was provided after every trial in both groups. Although this feedback was identical across groups and therefore does not explain the between-group difference, it may nevertheless have influenced learning across repeated trials. Finally, a design-related limitation is that only the Sonification group received an auditory preview of the correct sequence during observation. Accordingly, the present manipulation did not isolate the effects of concurrent auditory feedback during practice from those of an additional auditory encoding cue.

### 4.5 Conclusion

In this VR rhythm-game trajectory-learning task, congruent sonification during practice did not improve acquisition and was associated with reduced cue-reduced performance during silent memory-test trials after extended practice. The increased practice-to-test decrement in the Sonification group is consistent with accounts in which continuous augmented feedback functions as guidance and promotes cue dependency. These findings should be interpreted as specific to the present task structure and the present congruent parameter-mapping sonification, in which discrete left/center/right hand positions were mapped to discrete pitches, and should not be assumed to generalize directly to other tasks, movement classes, or sonification mappings.

## Data Availability

The raw data supporting the conclusions of this article will be made available by the authors, without undue reservation.
